# The Value of Routine Biochemical Tests in Discriminating Between Malignant and Benign Pancreatic Tumours

**DOI:** 10.1155/1991/17956

**Published:** 1991

**Authors:** L. Athlin, P-J Blind, S. Eriksson

**Affiliations:** Departments of Surgery and Geriatric Medicine Unioersity Hospital Umeå S-901 85 Sweden

## Abstract

The probability that routine hematological laboratory tests of liver and pancreatic function can discriminate between malignant and benign pancreatic tumours, incidentally detected during operation, was investigated. The records of 53 patients with a verified diagnosis of pancreatic carcinoma and 19 patients with chronic pancreatitis were reviewed with regard to preoperative total bilirubin, direct reacting bilirubin, alkaline phosphatase, glutamyltranspeptidase, aminotransferases, lactic dehydrogenase and amylase. Multivariate and discriminant analysis were performed to calculate the predictive value for cancer, using SYSTAT statistical package in a Macintosh II computer. Total and direct reacting bilirubin and glutamyltranspeptidase were significantly higher in patients with pancreatic carcinoma. However, only considerably increased levels of direct reating bilirubin were predictive of pancreatic carcinoma.


*HPB Surgery*, 1991, Vol. 4, pp. 147–155
Reprints available directly from the publisher
Photocopying permitted by license only

© 1991 Harwood Academic Publishers GmbH
Printed in the United Kingdom

### THE VALUE OF ROUTINE BIOCHEMICAL TESTS IN DISCRIMINATING BETWEEN MALIGNANT AND BENIGN PANCREATIC TUMOURS

L. ATHLIN, P-J BLIND and S. ERIKSSON*
*Departments of Surgery and *Geriatric Medicine, University Hospital, Umeå,, Sweden*

*(Received 15 January 1991)*

The probability that routine hematological laboratory tests of liver and pancreatic function can discriminate between malignant and benign pancreatic tumours, incidentally detected during operation, was investigated. The records of 53 patients with a verified diagnosis of pancreatic carcinoma and 19 patients with chronic pancreatitis were reviewed with regard to preoperative total bilirubin, direct reacting bilirubin, alkaline phosphatase, glutamyltranspeptidase, aminotransferases, lactic dehydrogenase and amylase. Multivariate and discriminant analysis were performed to calculate the predictive value for cancer, using SYSTAT statistical package in a Macintosh II computer. Total and direct reacting bilirubin and glutamyltranspeptidase were significantly higher in patients with pancreatic carcinoma. However, only considerably increased levels of direct reating bilirubin were predictive of pancreatic carcinoma.

KEY WORDS: Pancreas carcinoma, chronic pancreatitis, bilirubin, alkaline phosphatase, glutamyltranspeptidase, aminotransferases, lactic dehydrogenase, amylase

### INTRODUCTION

The diagnosis of pancreatic cancer is often difficult, even in the symptomatic patient[1]. Preoperatively the diagnosis usually is settled by means of computed tomography (CT) or ultrasound (US) and fine needle aspiration cytology (FNA)[2]. Pancreatic carcinoma is reported to be complicated by obstructive jaundice in 40–70% of cases[3]. However, specific symptoms are usually lacking which brings only about 10% of the patients with pancreatic carcinoma to curative resection at an early stage of the disease.

Also in patients with a pancreatic tumour incidentally detected peroperatively, the diagnosis of carcinoma or chronic pancreatitis is usually achieved by means of FNA or histopathologic examination (HPE). However, although the clinical findings point to a malignant tumour, no objective diagnostic verification is obtained in some cases since rapid staining or frozen section technique are not available. This raises the question, can routing biochemical serum assays of liver function, bile metabolism and amylase aid in discriminating between a benign and malignant pancreatic tumour?

Address correspondence to: Leif Athlin, Department of Surgery, University Hospital, S-901 85 UMEÅ, Sweden

### MATERIAL AND METHODS

The records of 72 consecutive patients who had a diagnosis of pancreatic carcinoma (53 patients) or chronic pancreatitis (19 patients) verified by FNA and/or HPE between March 1982 and May 1986 were reviewed. Patients with common duct stones were excluded. The median age of the 42 women and 30 men was 70 years (range 34–90 years).

TNM classification was used in the patients with carcinoma. Stage I was found in 9 patients, stage II in 9, stage III in 10 and stage IV in 18 patients. In 7 patients the stage of the tumour was not available. The tumour was resected with curative intent in seven patients.

The preoperative laboratory data collected from the records included total bilirubin, direct-reacting bilirubin, alkaline phosphatase (S-ALP), glutamyltranspeptidase (S-GT) aminotransferases, lactic dehydrogenase (LD) and amylase. The serum tests were performed according to the routines of the Clinical Chemistry Laboratory. The assays were unmodified during the study period.

### *Statistics*

Wilcoxon rank sum test[4] was used to compare laboratory data from the patients in the cancer group and the pancreatitis group. Factor analysis calculation was performed with varimax rotation to achieve and standardize an independent or orthogonal score for the predictors. This score was then used for calculations with the multiple general linear hypothesis program with models as described in text. All calculations were performed in a Macintosh II computer using SYSTAT[5].

### RESULTS

The results are summarized in Table 1.

### *Pancreatic Cancer*

Thirtyfive (66%) cancer patients had markedly elevated total bilirubin with mean value 210 μmol/L. Thirtyfour of these had the tumour in the head of pancreas. The mean value of total bilirubin in the whole group of 53 cancer patients was 148 μmol/L. The corresponding values of direct reacting bilirubin was 130 μmol/L and 82 μmol/L respectively. Also the mean values of S-ALP, S-GT, transferases and LD were above the upper reference limit in the total group of patients. Elevation of amylase with mean value 11,2 μmol/L was found in 7 patients.

Fortyfour patients had the tumour in the head of pancreas, 8 in the body or tail and in one the localization was not stated. Out of the 8 patients with the tumour in the body or tail of the pancreas, elevated values of bilirubin were found in one patient with liver metastases, S-ALP in 2, S-GT in 3, aminotransferases in 3 and LD in 4 patients. Amylase was normal in all these patients.

### *Chronic Pancreatitis*

Only three patients (16%) with chronic pancreatitis had elevated values of total

**Table 1** Preoperative biochemical serum assays of liver function, bile metabolism and amylase in patients with pancreatic cancer (A) or chronic pancreatitis (B). Values in brackets = upper reference limit; $n_1$ = number of tested patients; $n_2$ = number of patients with elevated values; % = per cent of patients with elevated values; $x_1$ = mean value in total number of tested patients; $x_2$ = mean value in patients with elevated values

| | *Tot bil (21 μmol/l)* | | *Dir bil (4,0 μmol/l)* | | *S-ALP (5,1 μkat/l)* | | *S-GT (0,52 μkat/l)* | | *ASAT (0,65 μkat/l)* | | *ALAT (0,65 μkat/l)* | | *LD (7,5 μkat/l)* | | *Amylase (4,0 μkat/l)* | |
|---|---|---|---|---|---|---|---|---|---|---|---|---|---|---|---|---|
| | *A* | *B* | *A* | *B* | *A* | *B* | *A* | *B* | *A* | *B* | *A* | *B* | *A* | *B* | *A* | *B* |
| $n_1$ | 53 | 19 | 51 | 18 | 53 | 18 | 51 | 18 | 53 | 18 | 53 | 18 | 51 | 18 | 41 | 10 |
| $n_2$ | 35 | 3 | 32 | 2 | 41 | 6 | 39 | 4 | 38 | 6 | 39 | 5 | 21 | 5 | 7 | 2 |
| % | 66 | 16 | 63 | 11 | 77 | 33 | 76 | 22 | 72 | 33 | 74 | 28 | 41 | 28 | 17 | 20 |
| $X_1$ | 148 | 17 | 82 | 4,3 | 14,8 | 10,5 | 5,1 | 1,9 | 2,10 | 0,93 | 2,9 | 1,27 | 8,1 | 7,1 | 3,4 | 3,6 |
| $X_2$ | 210 | 67 | 130 | 22 | 18,2 | 24,7 | 6,1 | 5,1 | 2,42 | 1,90 | 4,05 | 3,40 | 11 | 11,1 | 11,2 | 11,6 |

bilirubin. One of these (44 μmol/L) had pronounced cirrhotic liver disease without obstruction of extra hepatic ducts. Of the other two patients one was treated with an endoprosthesis (74 μmol/L) and one with a Whipple procedure (84 μmol/L). Direct reacting bilirubin values were elevated in two patients with pancreatitis. The mean values of S-ALP, S-GT and transferases were above the upper limit of normal in all patients with chronic pancreatitis. LD was elevated in 5 patients with mean 11,1 μmol/L but the mean value for the total group was within normal limits. Amylase was measured in only 10 patients with pancreatitis and was elevated in two of these (10,1 and 13,1 μmol/L). No false negative cancer diagnosis was made in any of the pancreatitis patients.

### *Statistical Analysis*

Mean values of total and direct reacting bilirubin and S-GT were higher in the patients with pancreatic carcinoma and the differences were statistically significant ($p<0.001$ and $p<0.05$, respectively). In 15 of the 19 patients (83%) with benign disease total and direct-reacting bilirubin below manufacturers upper reference limit. No significant differences were found in the other tests.

The multivariate analysis used for detection of predictive value of the biochemical parameters was based on data from 43 patients with complete data sets (34 with cancer and 9 with pancreatitis). Since the biochemical parameters used in this study were correlated they could not be used in a multivariate model for prediction without transformation. This was done with factor analysis where the first seven principal components explained 99,1% of the total variance and biochemical parameters were represented by one component except for direct reacting bilirubin + total bilirubin and ASAT + ALAT, which fell into the same components, respectively. The scores of each patient on these principal components were then used to calculate the independent predictive value for each biochemical parameter and the results from the multivariate calculations can be seen in Table 2. The results suggested that bilirubin may be of value in predicting cancer. Therefore, a discriminant analysis was performed where predictive value for cancer, after the a priori probability of cancer in this sample (0,79) was taken into account for each observed level of direct reacting bilirubin, was calculated and the results are displayed graphically in Figure 1. As can be seen only considerably increased levels of direct reacting bilirubin indicated cancer.

**Table 2** Predictive value for malignancy of seven orthogonal factor scores representing biochemical parameters and gender. The model is calculated as a multiple linear regression with F-ratio 0.54 (*p*-value 0.53)$R^2$ 15%

| | *Stand. regr. coefficient* | *p-value t-test* |
|---|---|---|
| Tot bil + dir bil | 0.29 | 0.08 |
| S-GT | 0.17 | 0.30 |
| ASAT + ALAT | 0.08 | 0.58 |
| Male gender | 0.08 | 0.62 |
| S-ALP | −0.07 | 0.66 |
| Amylase | −0.04 | 0.81 |
| LD | −0.03 | 0.84 |

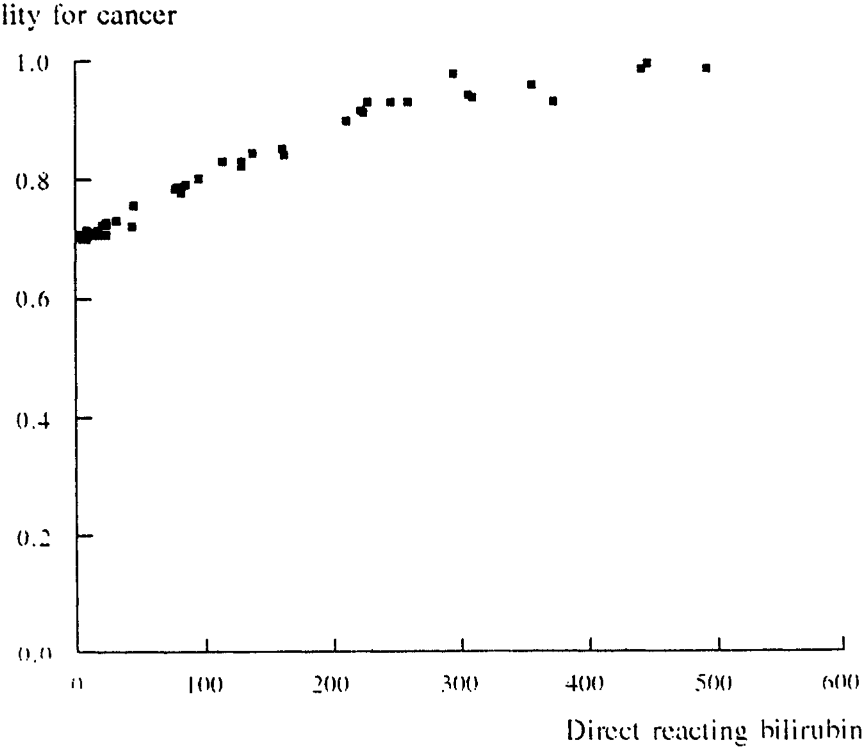

**Figure 1** Probability of cancer for observed levels of direct reacting bilirubin. The a priori probability of cancer in this series is 0.79.

### DISCUSSION

In some patients a pancreatic mass is incidentally found during operation. Preoperative FNA with rapid staining or HPE with frozen section may be used to obtain a morphologic diagnosis within a few minutes thereby guiding the choice of further surgery. However, these techniques are not available in all hospitals.

This retrospective study was undertaken to investigate the use of routine hematological laboratory tests to discriminate between malignant or benign pancreatic masses. We found that most of the tests were elevated in both the cancer and pancreatitis patients. Total and direct reacting bilirubin and GT were significantly higher in the patients with pancreatic carcinoma compared to those with chronic pancreatitis. In retrospective as well as in prospective studies on jaundiced patients a frequent cause for the hyperbilirubinemia seems to be pancreatic carcinoma and none, or only a few cases with chronic pancreatitis are reported[6–8]. This is in accordance with the results in the present study in which only 3 out of 19 patients with chronic pancreatitis had elevated bilirubin values. Furthermore, for one of the 3 cases the jaundice was caused by liver cirrhosis.

Fitzgerald *et al.*[9] evaluated the techniques currently employed in the diagnosis of pancreatic carcinoma. Of the hematologic routine tests, S-ALP had a high diagnostic accuracy while bilirubin was elevated in only half of the patients with pancreatic carcinoma. Lindberg[8], in a consecutive series of 144 patients with jaundice, found that in the differential diagnosis of pancreatic cancer and benign extrahepatic obstruction, bilirubin was the fourth and S-ALP the fifth most powerful indicant. Frebourg[10] did not find a diagnostic value for serum bilirubin in pancreatic cancer and Di Mango[11] could not demonstrate specificity for elevated values of S-ALP and bilirubin in excluding benign hepatic disorders.

In our study, factor and discriminant analysis demonstrated that only very high levels of direct reacting bilirubin may serve to predict pancreatic carcinoma.

In a retrospective study[12] on 868 patients with inflammatory pancreatic disease 3% were jaundiced due to chronic pancreatitis and extrahepatic obstruction and 1.4% required surgical decompression. However, not only the magnitude but also the duration and progression of the bilirubin levels should be taken into consideration in the diagnosis of a pancreatic tumour[13] since a transitory periductular pancreatic oedema may account for elevation of bilirubin even in some patients with chronic pancreatitis[12].

In the present study, S-ALP had elevated mean values in both the cancer patients and pancreatitis patients but did not differ between these groups. Seligson[14] found elevated S-ALP in 20 out of 24 patients with pancreatic malignancy which was significantly more common than in patients with chronic pancreatitis. However, elevation of S-ALP is not specific for pancreatic cancer since this can be found also in patients with other gastrointestinal tract cancers. Furthermore, biliary duct obstruction of any cause leads to elevation of S-ALP and may reflect that condition rather than be a marker of a specific disease of the pancreas islets[15].

Amylase was within the limits of the normal range in most of the patients in the present study which is in accordance with the results presented by Fitzgerald[9] who found elevated amylase activity in the serum of only 14% of patients with pancreatic cancer, in 31% of patients with other gastrointestinal cancers and in 9% of patients with chronic pancreatitis. Thus amylase seems of merit only in the diagnosis of acute pancreatitis[16].

The mean value of S-GT was above the upper reference limit in all patients in the present study, and significantly higher in the cancer patients compared to the pancreatitis patients. S-GT is distributed in different organs such as kidney, liver and pancreas. A specific S-GT seems to be present in pancreatic carcinoma cells[17]. Duct obstruction due to chronic pancreatitis increses S-GT[18]. Collins *et al.*[19] demonstrated a low sensitivity and specificity of S-GT in the differential diagnosis of jaundice.

In conclusion we found that jaundice was not frequent in patients with chronic pancreatitis. Although an elevation in routine hematological laboratory tests of liver and pancreatic function was common in the patients in the present study, only considerably raised levels of direct reacting bilirubin seemed to be of value in predicting cancer when a tumour was found in the head of pancreas. The discrepancy in the results of different studies evaluating diagnostic accuracy of routine biochemical tests in the diagnosis of pancreatic tumours may, to some extent, be explained by differences in laboratory techniques and variability in patients groups. In the individual patient, however, the final diagnosis and decision of resection should be achieved by mean of FNA or HPE.

*References*

1. Mackie, C.R., Cooper, M.J., Lewis, M.H. and Moossa, A.R. (1979) Nonoperative differentiation between pancreatic cancer and chronic pancreatitis. *Ann. Surg.*, **189**, 480–487
2. Ihse, I. and Isaksson, G. (1989) Preoperative and operative diagnosis of pancreatic cancer. *World J. Surg.*, **8**, 846–853
3. Brandabur, J.J., Kozarek, R.A., Ball, T.J., Hofer, B.O., Ryan, J.A., Traverso, L.W., Freeny, P.C. and Lewis, G.P. (1988) Nonoperative versus operative treatment of obstructive jaundice in pancreatic cancer: cost and survival analysis. *Am. J. Gastroenterol.*, **83**, 1132–1139
4. Siegel, S. (1956) Nonparametric statistics for the behavioural sciences. New York: McGraw-Hill
5. Wilkinson, L. (1986) SYSTAT. The system for statistics. Evanston, IL., USA Systat Inc.
6. Schenker, S., Balint, J. and Schiff, L. (1962) Differential diagnosis of jaundice: report of a prospective study of 61 proved cases. *Am. J. Dig. Dis.*, **7**, 449–463
7. Malchow-Möller, A., Matzon, P., Bjeeregaard, B., Hilden, J., Holst-Christensen, J., Staehr Johansen, T., Altman, L., Thomsen, C. and Juhl, E. (1981) Causes and characteristics of 500 consecutive cases of jaundice. *Scand. J. Gastroenterol.*, **16**, 1–6
8. Lindberg, G. (1982) Studies on diagnostic decision making in jaundice. Dissertation, Karolinska Institutet, Stockholm
9. Fitzgerald, P.J., Fortner, J.G., Watzon, R.C., Schwartz, M.K., Sherlock, P., Benua, R.S., Cubilla, A.L., Schottenfeld, D., Miller, D., Winawer, S.J., Lightdale, C.J., Leidner, S.D., Nisselbaum, J.S., Menendez-Botet, C.J. and Poleski, M.H. (1978) The value of diagnostic aids in detecting pancreas cancer. *Cancer*, **41**, 868–879
10. Frebourg, T., Bercoff, E., Manchon, N., Senant, J., Basuyau, J.P., Breton, P., Janvresse, A., Brunelle, P. and Boureille, J. (1988) The evaluation of CA 19–9 antigen level in the early detection of pancreatic cancer. *Cancer*, **62**, 2287–2290
11. Di Mango, E.P. (1988) Early diagnosis of chronic pancreatitis and pancreatic cancer. *Med. Clin. North Am.*, **72**, 979–992
12. Bradley, E.L. and Salam, A.A. (1978) Hyperbilirubinemia in inflammatory pancreatic disease: natural history and management. *Ann. Surg.*, **188**, 626–628
13. Kapur, B.M.L. (1986) Pancreaticogastrotomy in pancreatico-duodenal resection for ampullary carcinoma: experience in thirty-one cases. *Surgery*, **100**, 489–493
14. Seligison, U. and Söderlund, C. (1986) ERCP and serum alkaline phosphatase in pancreatic carcinoma. *Acta. Chir. Scand.*, **152**, 109–112
15. Kaplan, M.M. and Righetti, A. (1970) Induction of rat liver alkaline phosphatase: the mechanism of the serum elevation in bile duct obstruction. *J. Clin. Invest.*, **49**, 508–516
16. Ventrucci, M., Pezzili, R., Gullo, L., Plate, L., Sprovieri, G. and Barbara L. (1989) Role of serum pancreatic enzyme assays in diagnosis of pancreatic disease. *Dig. Dis. Sci.*, **34**, 39–45
17. Yamaguchi, N., Kawai, K. and Ashihara, T. (1986) Discrimination of gamma-glutamyltranspeptidase from normal and carcinomatous pancreas. *Clin. Chim. Acta*, **154**, 133–140
18. Graves, A.J., Holmquist, D.V. and Githens, S. (1986) Effect of duct obstruction on histology and on activities of gamma-glutamyltransferase, adenosine triphosphatase, alkaline phosphatase and amylase in rat pancreas. *Dig. Dis. Sci.*, **31**, 1254–1264
19. Collins, D., Goold, M.F., Rosalki, S.B., Mayne, P.D. and Foo, A.Y. (1987) Plasma intestinal alkaline phosphatase and intermediate molecular mass gamma-glutamyltransferase activities in the differential diagnosis of jaundice. *J. Clin. Pathol.*, **40**, 1252–1255

(Accepted S. Bengmark 15 January 1991)

### INVITED COMMENTARY

The differentiation between chronic pancreatitis and pancreatic cancer can be a difficult problem both preoperatively and intraoperatively, especially in an individual patient with a relatively early cancer. A large amount of confusion has evolved over the relationship between pancreatitis and pancreatic cancer mainly because the word "pancreatitis" is often used to connote several clinical, operativemacroscopic appearances, and histopathologic findings. The relationship between the two entities can be summarized as follows:

1. Patients with the acute or chronic form of pancreatitis of whatever etiology (gallstones, alcohol, etc.) are *not* at increased statistical risk from developing pancreatic cancer. The only notable rare exception is hereditary pancreatitis starting in early life where it is well documented that the patients are at high risk of developing pancreatic cancer in adult life.

2. Any patient with a pancreatic cancer or periampullary cancer (arising in the ampulla, duodenum, or distal common bile duct) may present with an acute episode or recurrent acute episode of documented clinical pancreatitis which is backed up by acute elevations of serum amylase, lipase, etc.

3. Histopathologic evidence of pancreatic inflammation, glandular destruction, and fibrosis, (i.e., pancreatitis), is invariably present in association with, and is probably induced by, any pancreatic tumor. This may cause diagnostic difficulties both pre-operatively and intraoperatively since the entire pancreatic mass may be much larger than the actual cancer. Further, needle biopsy of any mass will be subject to a varying degree of sampling error depending on the actual size and site of the tumor relative to the entire inflammatory mass.

Given the above considerations, any simple blood test that could indicate whether a neoplastic component is present in any pancreatic mass or not would solve a major clinical dilemma. Drs. Athlin and colleagues have employed multivariant and discriminant analysis on routine laboratory tests of liver and pancreatic function to discriminate between benign and malignant pancreatic lesions. Although the numbers of patients studies are small (53 patients with pancreatic carcinoma and 19 with chronic pancreatitis), they found that only considerably increased levels of direct reacting bilirubin were predictive of pancreatic carcinoma. What does this mean to the practicing physician? Simply stated, if a patient has a persistently and progressive evidence of biliary obstruction, the diagnosis is more likely to be pancreatic carcinoma than chronic pancreatitis. But the clinician must also remember that statistics do not necessarily apply to the individual patient. Thus, a patient with a small early pancreatic carcinoma may, on first presentation, have only a mildly elevated bilirubin. Alternatively, a patient with severe cicatrizing pancreatitis may have a highly elevated bilirubin level. Thus, the treating surgeon has to take the overall clinical picture into consideration and, if his index of suspicion for cancer is high enough, he may opt for a pancreatoduodenal resection irrespective of laboratory values or even negative needle biopsy results. Since the mortality of pancreatoduodenal resection is now in the region of one or two percent in expert hands, a case can be made for resecting any moderately suspicious lesion around the head of the pancreas irrespective of laboratory values or biopsy results. A thorough prospective study of various enzymes, human associated antigens, and pancreatic hormones, measured both in the systemic and portal venous systems have failed to show any possible useful addition to our diagnostic armamentarium [1].

### *References*

1. C.R. Mackie, A.R. Moossa, V.L. W. Go, G. Noble, G. Sizemore, M.J. Cooper, R.A.B. Wood, A.W. Ball, T. Waldmann, F. Gelder and A.H. Rubenstein (1980) Prospective evaluation of some candidate tumor markers in the diagnosis of pancreatic cancer. *Digestive Diseases and Sciences*, **25**, 161–172.

A.R. Moossa
Professor and Chairman
Department of Surgery
University of California
San Diego Medical Center
225 Dickinson Street
San Diego, California 92103
USA

